# A non-second-gradient model for nonlinear elastic bodies with fibre stiffness

**DOI:** 10.1038/s41598-023-33670-6

**Published:** 2023-04-21

**Authors:** M. H. B. M. Shariff, J. Merodio, R. Bustamante

**Affiliations:** 1 Department of Mathematics, Khalifa University of Science and Technology, Abu Dhabi, UAE; 2 Departamento de Matemática Aplicada a las TIC, ETS de Ingeniería de Sistemas Informáticos, Universidad Politécnica de Madrid, 28031 Madrid, Spain; 3 Departamento de Ingeniería Mecánica, Universidad de Chile, Beauchef 851, Santiago Centro, Santiago, Chile

**Keywords:** Aerospace engineering, Biomedical engineering, Civil engineering, Mechanical engineering, Engineering, Materials science, Biomaterials, Materials for devices, Soft materials, Structural materials

## Abstract

In the past, to model fibre stiffness of finite-radius fibres, previous finite-strain (nonlinear) models were mainly based on the theory of non-linear strain-gradient (second-gradient) theory or Kirchhoff rod theory. We note that these models characterize the mechanical behaviour of polar transversely isotropic solids with infinitely many purely flexible fibres with zero radius. To introduce the effect of fibre bending stiffness on purely flexible fibres with zero radius, these models assumed the existence of couple stresses (contact torques) and non-symmetric Cauchy stresses. However, these stresses are not present on deformations of actual non-polar elastic solids reinforced by finite-radius fibres. In addition to this, the implementation of boundary conditions for second gradient models is not straightforward and discussion on the effectiveness of strain gradient elasticity models to mechanically describe continuum solids is still ongoing. In this paper, we develop a constitutive equation for a non-linear non-polar elastic solid, reinforced by embedded fibers, in which elastic resistance of the fibers to bending is modelled via the classical branches of continuum mechanics, where the development of the theory of stresses is based on non-polar materials; that is, without using the second gradient theory, which is associated with couple stresses and non-symmetric Cauchy stresses. In view of this, the proposed model is simple and somewhat more realistic compared to previous second gradient models.

## Introduction

Fibre-reinforced composite materials have often been used in recent engineering applications. The rapid growth in manufacturing industries has led to the need for the improvement of materials in terms of strength, stiffness, density, and lower cost with improved sustainability. Fibre-reinforced composite materials have emerged as one of the materials possessing such improvement in properties serving their potential in a variety of applications^1–4^. The infusion of natural synthetic or natural fibers in the fabrication of composite materials has revealed significant applications in a variety of fields such as biomedical, automobile, mechanical, construction, marine and aerospace^5–8^. In biomechanics, some soft tissues can be modelled as fibre-reinforced composite materials^9,10^. In modern heavy engineering, the heavy traditional materials are gradually being replaced by fibre-reinforced polymer composite structures of lower weight and higher strength. These structures, such as railroads and bridges, are always under the action of dynamic moving loads caused by the moving vehicular traffic. Hence, in view of the above, a rigourous construction of a mechanical constitutive model, based on the sound theory of continuum mechanics, for non-polar fibre-reinforced solids, is paramount, and is of valuable interest in engineering designs and would find many practical applications.

The long history^11–13^ of mechanics of *non-polar* fiber-reinforced solids has, in general, significantly enriched and advanced the knowledge of solid mechanics. A boundary value problem for a non-polar elastic solid reinforced by (*finite radius*) fibres can be solved using the Finite Element Method (FEM), if small elements are permittable to mesh the fibres. If we treat the fibres to be an isotropic solid but have a different material properties from the matrix (material that is not attributable to the fibers) properties, we can use an inhomogeneous strain energy function

1 $$\begin{aligned} W(\lambda _1,\lambda _2,\lambda _3) \end{aligned}$$

in solving the FEM problem, where $\lambda _1,\lambda _2$ and $\lambda _3$ are the pricipal stretches. We note that, due to the finite radius of the fibres, bending resistance due to changes in the curvature for the fibres, is observed. However, if the fibre radius is significantly small, meshing the fibres and the matrix can be troublesome and hence it may not be possible to seek a boundary value solution via the FEM. To overcome this significantly small radius problem, a FEM solution can be obtained using a transversely elastic strain energy function^13^

2 $$\begin{aligned} W({\varvec{U}},{\varvec{a}}) \, \end{aligned}$$

where ${\varvec{U}}$ is the right-stretch tensor and ${\varvec{a}}$ is the unit preferred vector in the reference configuration. We note that this transversely isotropic model contains infinitely many purely flexible fibres with zero radius; hence this model cannot model elastic resistance due to changes in the curvature for the fibres. We emphasize that the Cauchy stress in both isotropic and transversely isotropic models is symmetric and this is actually observed in a non-polar solid in the absence of a couple stress. To model the effect of elastic resistance due to changes in the curvature for the fibres, recent models^14–17^ that are framed in the setting of the non-linear strain-gradient theory or Kirchhoff rod theory^18^, were developed. We note that these second-gradient models characterize the mechanical behaviour of (polar) transversely isotropic solids with infinitely many purely flexible fibres with zero radius. But, in order to simulate the effect of fibre bending stiffness on purely flexible fibres with zero radius, the second-gradient models introduce the existence of a couple stress and a non-symmetric Cauchy stress in the constitutive equations; we must emphasize that both of these stresses are not present on deformations of actual non-polar elastic solids reinforced by finite-radius fibres. In general, higher gradient elasticity models are used to describe mechanical structures at the micro-and nano-scale or to regularize certain ill-posed problems by means of these higher gradient contributions. Discussion on the effectiveness of higher gradient elasticity models to mechanically describe continuum solids is still ongoing^19–21^.

Hence, the objective of this paper is to propose an approximate model to simulate the mechanical behaviour of actual non-polar elastic solids reinforced by finite- radius fibres, where the Cauchy stress is symmetric and fibre bending resistance is caused by changes in curvature of the fibres. We focus on changes in fibre curvature, since in composite solids, these changes play an important role in the mechanical behaviour of solids. Since our model contains infinitely many fibres with zero radius, we exclude the effects due to fibre ’twist’. In fact Spencer and Soldatos^17^ stated that

*“In doing this, we exclude effects due to fibre ’splay’ and fibre ’twist’, both of which feature in liquid crystal theory, but it is plausible that in fibre composite solids the major factor is fibre curvature.”*

Our proposed model does not require couple stresses (which are not observed in actual non-polar elastic solids reinforced by finite-radius fibres) to describe elastic resistance of the fibers to bending.

Spectral approach^14,22^ is used in the modelling and this is preliminary described in Sects. “Preliminaries” and “Strain energy function”, where in Sect. “Strain energy function” a strain energy function contains a vector that governs the changes in the fibre curvature. A prototype of the strain energy is given in Sect. “Strain energy prototype” and boundary value problems to study the effect of fibre bending resistance are presented in Sect. “Boundary value problem”.

**Figure 1:**
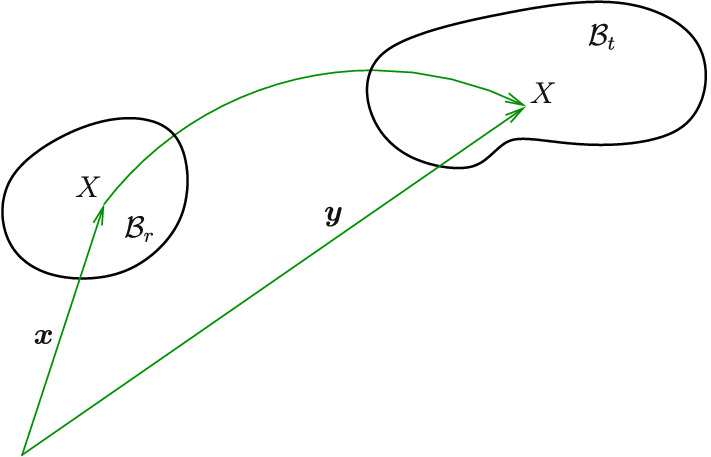
Deformation due to application of boundary displacement and boundary traction. $B_r$ is the reference (undeformed) configuration, $B_t$ is the current configuration, ${\varvec{x}}$ and ${\varvec{y}}$ are, respectively, the position vectors of *X* in the reference and current configurations, where *X* represents a generic particle of the solid body.

## Preliminaries

In this communication, all subscripts i, j and k take the values 1,2,3, unless stated otherwise. In terms of spectral invariants, the deformation gradient ${\varvec{F}}$ is described via

3 $$\begin{aligned} {\varvec{F}}(\lambda _i,{\varvec{v}}_i,{\varvec{u}}_i)={\displaystyle \frac{\partial {\varvec{y}}}{\partial {\varvec{x}}}} = \sum _{i=1}^3 \lambda _i {\varvec{v}}_i\otimes {\varvec{u}}_i \,, \end{aligned}$$

where ${\varvec{y}}$ and ${\varvec{x}}$ denote, respectively, the position vectors of a solid body particle in the current and reference configurations (see Fig. 1 ) ; $\lambda _i$ is a principal stretch, ${\varvec{v}}_i$ is an eigenvector of the left stretch tensor ${\varvec{V}}= {\varvec{F}}(\lambda _i,{\varvec{v}}_i,{\varvec{v}}_i)$ and ${\varvec{u}}_i$ is an eigenvector of the right- stretch tensor ${\varvec{U}}= {\varvec{F}}(\lambda _i,{\varvec{u}}_i,{\varvec{u}}_i)$. Note that the right Cauchy-Green tensor ${\varvec{C}}= {\varvec{F}}(\lambda _i^2,{\varvec{u}}_i,{\varvec{u}}_i)$ and the rotation tensor ${\varvec{R}}= {\varvec{F}}(\lambda _i=1,{\varvec{v}}_i,{\varvec{u}}_i)$, where ${\varvec{F}}={\varvec{R}}{\varvec{U}}$. We only consider incompressible elastic solids, where $\det {\varvec{F}}=1$, $\det$ indicates the determinant of a tensor and the effect of body forces is assumed negligible. The summation convention is not used here.

## Strain energy function

To model the kinematics of the embedded fibers, we assume the body, regarded as a homogenized continuum consisting of matrix material and fibers together. We model this material by considering a transversely elastic solid with the preferred unit directions ${\varvec{a}}({\varvec{x}})$ in the reference configuration and these preferred directions becomes the vector

4 $$\begin{aligned} {\varvec{b}}= {\varvec{F}}{\varvec{a}}= \varrho {\varvec{f}}\,, \,\varrho = \sqrt{{\varvec{a}}\cdot {\varvec{C}}{\varvec{a}}} > 0 \,, \end{aligned}$$

in the current configuration, where ${\varvec{f}}$ is a unit vector. In our proposed model, the directional derivative of the fibre unit vector in the fibre direction, i.e.,

5 $$\begin{aligned} {\varvec{c}}= {\displaystyle \frac{\partial {\varvec{f}}}{\partial {\varvec{x}}}} {\varvec{a}}\,, \end{aligned}$$

plays an important role in modelling elastic resistance due to changes in curvature for the fibres. In view of this we endow a vector ${\varvec{d}}$ associated with ${\varvec{c}}$ (we will make the association clear later) in (5), which is independent of ${\varvec{F}}$, i.e.^14,15^

6 $$\begin{aligned} {\varvec{d}}= {\displaystyle \frac{1}{\iota }}{\varvec{\Lambda }}{\varvec{a}}- {\displaystyle \frac{1}{\iota ^3}}({\varvec{a}}\cdot {\varvec{\Lambda }}{\varvec{a}}){\bar{{\varvec{C}}}}{\varvec{a}}\,, \,\iota = \sqrt{{\varvec{a}}\cdot {\bar{{\varvec{C}}}}{\varvec{a}}} \,, \end{aligned}$$

where

7 $$\begin{aligned} {\bar{{\varvec{C}}}} ={\bar{{\varvec{F}}}}^T{\bar{{\varvec{F}}}} \,, \,{\varvec{\Lambda }}= {\bar{{\varvec{F}}}}^T{\varvec{G}}- {\displaystyle \frac{\partial {\varvec{a}}}{\partial {\varvec{x}}}} \,, \,{\varvec{G}}= {\displaystyle \frac{\partial {\bar{{\varvec{F}}}}{\varvec{a}}}{\partial {\varvec{x}}}} \,, \end{aligned}$$

**Figure 2:**
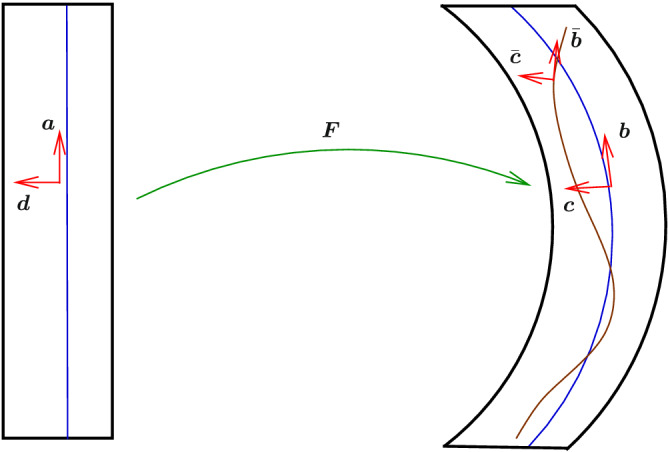
Geometric meaning of directional derivative vectors: ${\varvec{F}}\ne {\bar{{\varvec{F}}}}$,  ${\varvec{a}}\cdot {\varvec{d}}={\varvec{b}}\cdot {\varvec{c}}= {\bar{{\varvec{b}}}}\cdot {\bar{{\varvec{c}}}} = 0$, ${\bar{{\varvec{b}}}} = {\bar{{\varvec{F}}}}{\varvec{a}}=\iota {\bar{{\varvec{f}}}}$,  and  ${\bar{{\varvec{c}}}} = {\displaystyle \frac{\partial {\bar{{\varvec{f}}}}}{\partial {\varvec{x}}}}{\varvec{a}}$.

${\bar{{\varvec{F}}}}({\varvec{x}})$ is the deformation tensor independent of ${\varvec{F}}$, i.e., ${\varvec{d}}$ is not embedded in the matrix, and so in general its image ${\bar{{\varvec{F}}}}^{-T}{\varvec{d}}$ in the current configuration is not directly connected to the deformation of the matrix. Clearly from (6), we have ${\varvec{d}}\cdot {\varvec{a}}=0$ (See Fig. 2 for geometric interpretation). If we let ${\bar{{\varvec{F}}}}={\varvec{F}}$, we then have the association ${\varvec{c}}= {\varvec{F}}^{-T}{\varvec{d}}$^14,15^. To facilitate the process of modelling, we express the vector

8 $$\begin{aligned} {\varvec{d}}= \rho {\varvec{k}}\,, \,\rho = \sqrt{{\varvec{d}}\cdot {\varvec{d}}} \,, \end{aligned}$$

where ${\varvec{k}}$ is a unit vector with the property ${\varvec{a}}\cdot {\varvec{k}}=0$. To model elastic resistance due to changes in curvature of the fibres, we assume the objective strain energy

9 $$\begin{aligned} W = {W}_{(a)}({\varvec{U}},{\varvec{a}},{\varvec{k}},\rho ) = {W}_{(a)}\left( {\varvec{Q}}{\varvec{U}}{\varvec{Q}}^T,{\varvec{Q}}{\varvec{a}},{\varvec{Q}}{\varvec{k}},\rho \right) \,, \end{aligned}$$

for every rotation tensor ${\varvec{Q}}$. Following, the work of Shariff^22,23^, *W* can be characterised by the spectral invariants

10 $$\begin{aligned} \lambda _i \, \,a_i = {\varvec{a}}\cdot {\varvec{u}}_i, \,b_i ={\varvec{k}}\cdot {\varvec{u}}_i \,, \,\sum _{i=1}^3 a_i^2 = 1 \,, \,\sum _{i=1}^3 b_i^2 = 1 \end{aligned}$$

and the scaler $\rho$, where $\lambda _i$ and ${\varvec{u}}_i$ are, repectively, the eigenvalues and eigenvectors of ${\varvec{U}}$. Hence, we can express

11 $$\begin{aligned} W = {W}_{(a)}(\lambda _i,a_i,b_i,\rho ) \,, \end{aligned}$$

taking note the ${W}_{(a)}$ must satisfy the *P*-property described in^24^ associated with the coalescence of principal stretches $\lambda _i$. In view that *W* should be independent of the sign of ${\varvec{a}}$ and ${\varvec{d}}$, we express

12 $$\begin{aligned} W = {W}_{(s)}(\lambda _i,\alpha _i,\beta _i,\rho ) \,, \,\alpha _i=a_i^2 \,, \,\beta _i = b_i^2 \,. \end{aligned}$$

### Spectral derivative components

The evaluation of stress tensors requires spectral the Lagrangian spectral tensor components of ${\displaystyle \frac{\partial W}{\partial {\varvec{C}}}}$ i.e.,

13 $$\begin{aligned} \left( {\displaystyle \frac{\partial W}{\partial {\varvec{C}}}} \right) _{ii}= & {} {\displaystyle \frac{1}{2\lambda _i}}{\displaystyle \frac{\partial {W}_{(s)}}{\partial \lambda _i}} \,, \end{aligned}$$

14 $$\begin{aligned} \left( {\displaystyle \frac{\partial W}{\partial {\varvec{C}}}} \right) _{ij}= & {} {\displaystyle \frac{1}{(\lambda _i^2 -\lambda _j^2)}} \left\{ \left( {\displaystyle \frac{\partial {W}_{(s)}}{\partial \alpha _i}} - {\displaystyle \frac{\partial {W}_{(s)}}{\partial \alpha _j}}\right) a_ia_j + \left( {\displaystyle \frac{\partial {W}_{(s)}}{\partial \beta _i}} - {\displaystyle \frac{\partial {W}_{(s)}}{\partial \beta _j}}\right) b_ib_j \right\} \,, \quad i\ne j \,. \end{aligned}$$

The Eulerian spectral components of the Cauchy stress ${\varvec{T}}$ for an incompressible body with respect to the spectral Eulerian basis $\{ {\varvec{v}}_1,{\varvec{v}}_2,{\varvec{v}}_3\}$ are

15 $$\begin{aligned} \tau _{ii}=\lambda _i{\displaystyle \frac{\partial {W}_{(s)}}{\partial \lambda _i}} -p \,, \,\tau _{ij}= 2\lambda _i\lambda _j \left( {\displaystyle \frac{\partial W}{\partial {\varvec{C}}}} \right) _{ij} \,, \,i\ne j \,. \end{aligned}$$

## Strain energy prototype

In this paper, following the work of Shariff^22^, we nominate a prototype strain energy function that satisfy the *P*-property. We emphasize that our proposed non-linear strain energy function is consistent with the theory of infinitesimal elasticity. To ensure this consistency, we start our non-linear prototype construction via developing its infinitesimal strain energy counterpart.

### Infinitesimal strain energy function

Before we construct strain energy prototypes for finite strain deformation, we give a brief description on infinitesimal elasticity. When the gradient of the displacement field ${\varvec{u}}$ is very small

16 $$\begin{aligned} \Vert {\varvec{F}}-{\varvec{I}}\Vert = \Vert {\displaystyle \frac{\partial {\varvec{u}}}{\partial {\varvec{x}}}} \Vert = O(e) \,, \end{aligned}$$

where $\Vert \bullet \Vert$ is an appropriate norm and the magnitude of *e* is much less than unity. Up to *O*(*e*),

17 $$\begin{aligned} {\varvec{U}}- {\varvec{I}}= {\varvec{E}}\,, \end{aligned}$$

where ${\varvec{E}}$ is the infinitesimal strain. The most general quadratic form of the strain energy function is

18 $$\begin{aligned} W= {W}_{(T)} + {W}_{(\Lambda )} \,, \end{aligned}$$

where

19 $$\begin{aligned} {W}_{(T)}= & {} \mu \text{ tr }{\varvec{E}}^2 + 2\mu _1 {\varvec{a}}\cdot {\varvec{E}}^2{\varvec{a}}+ {\displaystyle \frac{\kappa _1}{2}}({\varvec{a}}\cdot {\varvec{E}}{\varvec{a}})^2 \,, \end{aligned}$$

20 $$\begin{aligned} {W}_{(\Lambda )}= & {} 2\mu _2 \rho ^2 {\varvec{k}}\cdot {\varvec{E}}^2{\varvec{k}}+ {\displaystyle \frac{\kappa _2}{2}} \rho ^4 ({\varvec{k}}\cdot {\varvec{E}}{\varvec{k}})^2 + \kappa _3 \rho ^2 ({\varvec{a}}\cdot {\varvec{E}}{\varvec{a}})( {\varvec{k}}\cdot {\varvec{E}}{\varvec{k}}) \,, \end{aligned}$$

where $\mu , \mu _1, \mu _2, \kappa _1, \kappa _2,\kappa _3$ are ground-state material constants and their limitations are given in Online Appendix A.

### Finite strain

We propose finite-strain energy function that is consistent with its infinitesimal counterpart. This can be easily done, following the work of Shariff^22^, by extending the above infinitesimal strain energy function using spectral generalized strains for finite deformations. The proposed strain energy function is

21 $$\begin{aligned} W= {W}_{(A)} + {W}_{(\Lambda )} \,, \end{aligned}$$

where

22 $$\begin{aligned} {W}_{(T)}= & {} \mu \sum _{i=1}^3 r_1^2(\lambda _i) + 2\mu _1\sum _{i=1}^3 \alpha _i r_2^2(\lambda _i) + {\displaystyle \frac{\kappa _1}{2}} \left( \sum _{i=1}^3 \alpha _i r_3(\lambda _i)\right) ^2 \,, \end{aligned}$$

23 $$\begin{aligned} {W}_{(\Lambda )}= & {} 2\mu _2\rho ^2 \sum _{i=1}^3 \beta _i r_4^2(\lambda _i) + {\displaystyle \frac{\kappa _2}{2}}\rho ^4 \left( \sum _{i=1}^3 \beta _i r_5(\lambda _i)\right) ^2 + \kappa _3 \rho ^2 \left[ \sum _{i=1}\alpha _i r_6(\lambda _i)\right] \left[ \sum _{i=1}^3 \beta _i r_7(\lambda _i)\right] \,, \end{aligned}$$

with the properties^22^

24 $$\begin{aligned} r_\alpha (1) =0 \,, \,r_\alpha '(1) = 1 \,, \,\alpha =1,2,\ldots 7 \,. \end{aligned}$$

We could also include the following property, when appropriate, $r_\alpha$ to represent physical strain measures with the extreme deformation values

25 $$\begin{aligned} r_\alpha (\lambda _i \rightarrow \infty ) =\infty \,, \,r_\alpha (\lambda \rightarrow 0) = -\infty \,. \end{aligned}$$

We could easily extend (21) to (23) to construct a more general strain energy function (see for example^22^), but the strain energy function proposed in the Section should suffice to illustrate our model.

## Boundary value problem

To illustrate our theory, we consider two simple deformations, pure bending and finite torsion of a right circular cylinder, where their displacements are known. For boundary value problems, where the displacements are unknown, the construction of solutions are described in Online Appendix B.

To plot the results in this section, for simplicity, we use

26 $$\begin{aligned} r_\alpha (x) = \ln (x) \,, \,\alpha =1,2,\ldots 7 \,, \end{aligned}$$

and the ground-state values

27 $$\begin{aligned} \mu =5\text{ kPa } \,, \,\mu _1=80 \text{ kPa } \,, \,\kappa _1 = 0 \,, \end{aligned}$$

are those associated with skeletal muscle tissue^10,25^. Since our model is new and there are no experimental values for the following bending stiffness ground-state constants, we use the ad hoc values

28 $$\begin{aligned} \mu _2=10.0 \text{ kPa } \,, \,\kappa _1=\kappa _2=0 \,, \,\kappa _3=-100 \text{ kPa } \,. \end{aligned}$$

to plot the graphs. Take note that the above values satisfy the restrictions given in Appendix A.

**Figure 3:**
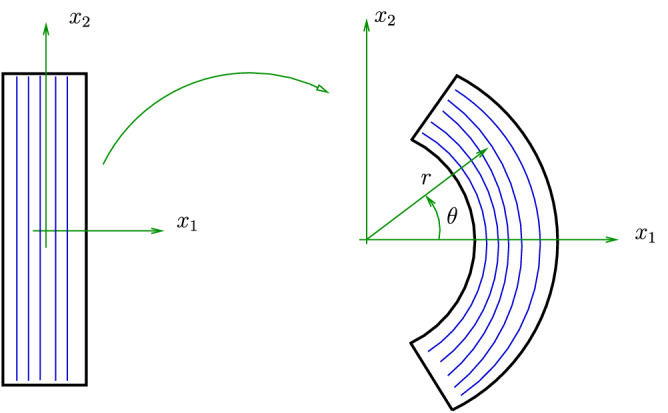
Bending of a rectangular block into a sector of a cylindrical tube.

### Pure bending

Consider the problem of pure bending in plane strain, depicted in Fig. 3, in which a rectangular slab of incompressible material is bent into a sector of a circular annulus defined by

29 $$\begin{aligned} r=r(x_1) \,, \,\theta = \theta (x_2) \,, \,z=x_3 \,, \,0 \le x_1 \le B \,, \,-L \le x_2 \le L \,, \,-H \le x_3 \le H \,, \end{aligned}$$

where $(r,\theta ,z)$ is the cylindrical polar coordinate for the current configuration and $(x_1,x_2,x_3)$ is the Cartesian referential coordinate with the basis $\{ {\varvec{g}}_1 , {\varvec{g}}_2, {\varvec{g}}_3 ={\varvec{e}}_z \}$.

The formula employed here could be used to compare our theory with experiment (for example, a three point bending test experiment described in reference^26^).

The deformation tensor has the form

30 $$\begin{aligned} {\varvec{F}}= r' {\varvec{e}}_r\otimes {\varvec{g}}_1 + r\theta ' {\varvec{e}}_\theta \otimes {\varvec{g}}_2 + {\varvec{e}}_z\otimes {\varvec{g}}_3 \,. \end{aligned}$$

From the incompressibility condition $\det {\varvec{F}}=1$ and the boundary conditions $\theta (0)=0$ and $r(A)=a$ we obtain

31 $$\begin{aligned} r^2 - a^2 = 2\chi x_1 \,, \,\theta = {\displaystyle \frac{x_2}{\chi }} \,, \,\chi = {\displaystyle \frac{b^2-a^2}{2B}} > 0\,, \end{aligned}$$

where $r(B) = b$. Hence, in view of (3), (30) and (31), we have

32 $$\begin{aligned} \lambda _1 = {\displaystyle \frac{\chi }{r}} \,, \,\lambda _2 = {\displaystyle \frac{r}{\chi }} \,, \,\lambda _3 = 1 \, \end{aligned}$$

and the spectral basis vectors are ${\varvec{u}}_i={\varvec{g}}_i$, ${\varvec{v}}_1={\varvec{e}}_r$, ${\varvec{v}}_2={\varvec{e}}_\theta$ and ${\varvec{v}}_3={\varvec{e}}_z$.

In this section we study the case ${\varvec{a}}={\varvec{g}}_2$, hence, $a_1=a_3=0$ and $a_2=1$. If we let ${\bar{{\varvec{F}}}}={\varvec{F}}$, we get

33 $$\begin{aligned} {\varvec{k}}=-{\varvec{g}}_1 \,, \,\rho ={\displaystyle \frac{1}{r}} \,, \,b_1= -1 \,, \,b_2=b_3=0 \,. \end{aligned}$$

The strain energy function is simplified, i.e.

34 $$\begin{aligned} {W}_{(T)}= & {} \mu \sum _{i=1}^3 r_1^2(\lambda _i) + 2\mu _1 r_2^2(\lambda _2) + {\displaystyle \frac{\kappa _1}{2}}r_3^2(\lambda _2) \,, \nonumber \\ {W}_{(\Lambda )}= & {} 2\rho ^2 \mu _2 r_4^2(\lambda _1) + \rho ^4 {\displaystyle \frac{\kappa _2}{2}}r_5^2(\lambda _1) + \rho ^2\kappa _3r_6(\lambda _2)r_7(\lambda _1) \,, \,W = {W}_{(T)} + {W}_{(\Lambda )} \,. \end{aligned}$$

The non-zero Cauchy stress components simply becomes

35 $$\begin{aligned} \sigma _i = \lambda _i\left( {\displaystyle \frac{\partial {W}_{(T)}}{\partial \lambda _i}} + {\displaystyle \frac{\partial {W}_{(\Lambda )}}{\partial \lambda _i}}\right) - p \,, \end{aligned}$$

where $\sigma _1=\sigma _{rr}$, $\sigma _2=\sigma _{\theta \theta }$ and $\sigma _3=\sigma _{zz}$ are cylindrical components of the Cauchy stress. Since $\sigma _i$ depends only on *r*, the equilibrium equation simply becomes

36 $$\begin{aligned} {\displaystyle \frac{d \sigma _{rr}}{d r}} + {\displaystyle \frac{1}{r}} (\sigma _{rr} - \sigma _{\theta \theta }) = 0 \,. \end{aligned}$$

If we assume that $\sigma _{rr} = 0$ at $r = b$, we then have

37 $$\begin{aligned} \sigma _{rr} = - \int _r^b G(y) \, dy \,, \,rG(r) = \lambda _2{\displaystyle \frac{\partial W}{\partial \lambda _2}} - \lambda _1{\displaystyle \frac{\partial W}{\partial \lambda _1}} \,. \end{aligned}$$

Hence, we can evaluate

38 $$\begin{aligned} p = \lambda _1 {\displaystyle \frac{\partial W}{\partial \lambda _1}} + \int _r^b G(y) \, dy \end{aligned}$$

and with the above expression for *p* we obtain the stress-strain relations for $\sigma _{\theta \theta }$ and $\sigma _{zz}$. The bending moment ${\mathcal {M}}$, and the normal force ${\mathcal {N}}$, per unit length in the $x_3$ direction, and applied to a section of constant $\theta$, are

39 $$\begin{aligned} {\mathcal {M}} = \int _a^b r\sigma _{\theta \theta } \, dr \,, \,{\mathcal {N}}= \int _a^b \sigma _{\theta \theta } dr \,. \end{aligned}$$

**Figure 4:**
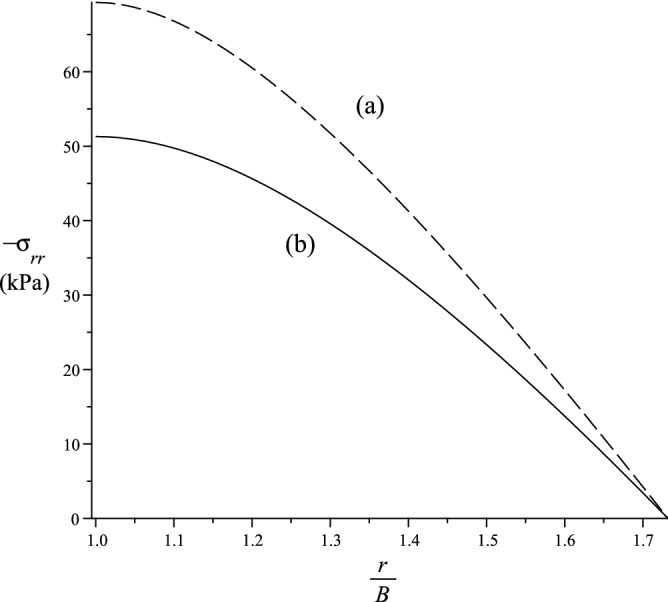
Radial behaviour of stress $\sigma _{rr}$. (**a**) Elastic solid with fibre bending resistance. (**b**) Elastic solid with no fibre bending resistance.

**Figure 5:**
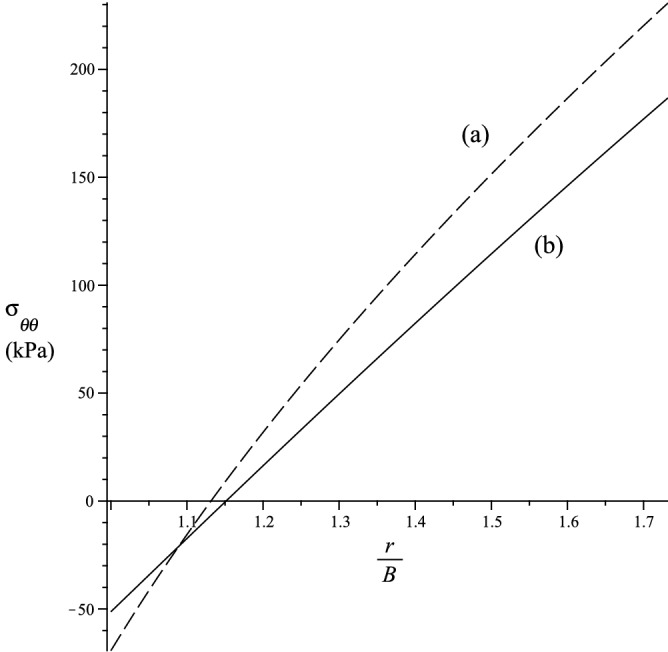
Radial behaviour of stress $\sigma _{\theta \theta }$. (**a**) Elastic solid with fibre bending resistance. (**b**) Elastic solid with no fibre bending resistance.

In Figs. 4 and 5, the behaviours of, respectively, the radial and hoop stresses are depicted using ${\displaystyle \frac{\chi }{B}}=1$ and the material is deformed to ${\displaystyle \frac{a}{B}}=1$. It is clear from these figures the magnitude of the stresses is higher for an elastic solid with fibre bending resistance than for a solid with perfectly flexible fibres.

The ${\mathcal {M}}$ values for a material with and without fibre bending resistance are, respectively, 46.44514245 kPaM$^2$ and 35.55851694 kPaM$^2$. The ${\mathcal {N}}$ values for a material with and without fibre bending resistance are, respectively, 30.58637503 kPaM and 23.29228593 kPaM. Hence, bending stiffness increases the magnitude of ${\mathcal {M}}$ and ${\mathcal {N}}$.

## Torsion and extension of a cylinder

In this section we consider an incompressible thick-walled circular cylindrical annulus with the initial geometry

40 $$\begin{aligned} 0 \le R \le A, \quad 0\le \Theta \le 2\pi , \quad 0\le Z \le L, \end{aligned}$$

where *R*, $\Theta$ and *Z* are reference polar coordinates with the corresponding basis $B_R=\{ {\varvec{E}}_R,{\varvec{E}}_\Theta ,{\varvec{E}}_Z \}$. The boundary value problem illustrated here could be used in an experiment (see, for example, reference^27^) to verify our theoretical predictions.

**Figure 6:**
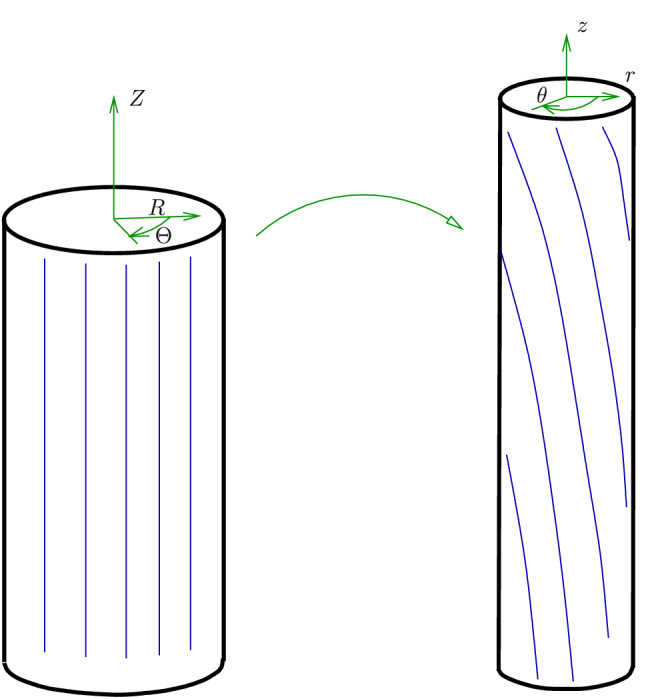
Torsion and extension of a cylinder.

The deformation is depicted in Fig. 6 and is described by

41 $$\begin{aligned} r=\lambda _z^{-\frac{1}{2}}R, \quad \theta = \Theta + \lambda _z\tau Z, \quad z=\lambda _z Z, \end{aligned}$$

where $\tau$ is the amount of torsional twist per unit deformed length and $\lambda _z$ is the axial stretch. In the above formulation, *r*, $\theta$ and *z* are cylindrical polar coordinates in the deformed configuration with the corresponding basis $B_C=\{ {\varvec{e}}_r,{\varvec{e}}_\theta ,{\varvec{e}}_z \}$. Here, we have allowed ${\varvec{e}}_r={\varvec{E}}_R$, ${\varvec{e}}_\theta ={\varvec{E}}_\Theta$ and ${\varvec{e}}_z={\varvec{E}}_Z$. The deformation gradient is

42 $$\begin{aligned} {\varvec{F}}= \lambda _z^{-1/2} {\varvec{e}}_r\otimes {\varvec{E}}_R + \lambda _z^{-1/2} {\varvec{e}}_\theta \otimes {\varvec{E}}_\Theta + \lambda _z\gamma {\varvec{e}}_\theta \otimes {\varvec{E}}_Z + \lambda _z {\varvec{e}}_z\otimes {\varvec{E}}_Z \,, \end{aligned}$$

where $\gamma =r\tau$ and in this paper, we only consider $\lambda _z \ge 1$. The Lagrangian principal directions are:

43 $$\begin{aligned} {\varvec{u}}_1 ={\varvec{E}}_R \,, \,{\varvec{u}}_2 =c {\varvec{E}}_\Theta + s{\varvec{E}}_Z \,, \,{\varvec{u}}_3 =-s{\varvec{E}}_\Theta + c{\varvec{E}}_Z \,, \end{aligned}$$

where

44 $$\begin{aligned} c = \cos (\phi )=\frac{2}{\sqrt{2({\hat{\gamma }}^2+4)+ 2{\hat{\gamma }}\sqrt{{\hat{\gamma }}^2+4}}}, \quad s= \sin (\phi )=\frac{{\hat{\gamma }}+\sqrt{{\hat{\gamma }}^2+4}}{\sqrt{2({\hat{\gamma }}^2+4)+ 2{\hat{\gamma }}\sqrt{{\hat{\gamma }}^2+4}}}, \end{aligned}$$

with

45 $$\begin{aligned} \frac{\pi }{4} \le \frac{\pi -\tan ^{-1}\left( \frac{1}{\sqrt{\lambda _z^3-1}}\right) }{2} \le \phi < \frac{\pi }{2} \,, \,{\hat{\gamma }} = \frac{\lambda _z^3\gamma ^2 +\lambda _z^3-1}{\lambda _z^{\frac{3}{2}}\gamma } \ge 0 \,, \,c^2-s^2 = -{\hat{\gamma }} cs \,. \end{aligned}$$

In the case of pure torsion, $\lambda _z=1$ and we have ${\hat{\gamma }}=\gamma$. The principal stretches for a combined extension and torsion deformation are

46 $$\begin{aligned} \lambda _1 =\frac{1}{\lambda _z^{\frac{1}{2}}} \,, \,\lambda _2 =\sqrt{\frac{1}{\lambda _z} +\frac{s\gamma \sqrt{\lambda _z}}{c}}\,, \,\lambda _3 =\sqrt{\frac{1}{\lambda _z} -\frac{c\gamma \sqrt{\lambda _z}}{s}}. \end{aligned}$$

In this section we consider the case when ${\varvec{a}}={\varvec{E}}_z$, hence, $a_1=0$, $a_2=s$ and $a_3=c$. If we let ${\bar{{\varvec{F}}}}={\varvec{F}}$ and using

47 $$\begin{aligned} \text{ Grad }{\varvec{b}}= {\displaystyle \frac{\partial {\varvec{b}}}{\partial R}}\otimes {\varvec{E}}_R + {\displaystyle \frac{1}{R}} {\displaystyle \frac{\partial {\varvec{b}}}{\partial \Theta }}\otimes {\varvec{E}}_\Theta + {\displaystyle \frac{\partial {\varvec{b}}}{\partial Z}}\otimes {\varvec{E}}_Z \,, \end{aligned}$$

we obtain

48 $$\begin{aligned} {\varvec{k}}= -{\varvec{E}}_R \,, \,\rho ={\displaystyle \frac{\lambda _z^3\gamma \tau }{\sqrt{\lambda _z^2(1+\gamma ^2)}}} \,, \,b_1=-1 \,,\,b_2=b_3=0 \,. \end{aligned}$$

The strain energy function then takes the form

49 $$\begin{aligned} {W}_{(T)}= & {} \mu \sum _{i=1}^3 r_1^2(\lambda _i) + 2\mu _1 \left[ s^2r_2^2(\lambda _2)+c^2r_2^2(\lambda _3)\right] + {\displaystyle \frac{\kappa _1}{2}}\left[ s^2r_3(\lambda _2)+c^2r_3(\lambda _3)\right] ^2 \,, \nonumber \\ {W}_{(\Lambda )}= & {} 2\rho ^2\mu _2r_4^2(\lambda _1) + \rho ^4 {\displaystyle \frac{\kappa _2}{2}}r_5^2(\lambda _1) + \rho ^2\kappa _3\left[ s^2r_6(\lambda _2)+c^2r_6(\lambda _3)\right] r_7(\lambda _1) \,. \end{aligned}$$

The Cauchy stress

50 $$\begin{aligned} {\varvec{T}}= 2{\varvec{F}}{\displaystyle \frac{\partial W}{\partial {\varvec{C}}}}{\varvec{F}}^T - pI \,. \end{aligned}$$

In view of ${\varvec{a}}\equiv [0,0,1]^T$, we have $a_1=0$, $a_2=s$ and $a_3=c$ and

51 $$\begin{aligned} {\varvec{T}}= \sigma _{rr} {\varvec{e}}_r\otimes {\varvec{e}}_r + \sigma _{\theta \theta }{\varvec{e}}_\theta \otimes {\varvec{e}}_\theta + \sigma _{zz} {\varvec{e}}_z\otimes {\varvec{e}}_z + \sigma _{z\theta }({\varvec{e}}_z\otimes {\varvec{e}}_\theta + {\varvec{e}}_\theta \otimes {\varvec{e}}_z) \,, \end{aligned}$$

where

52 $$\begin{aligned} \sigma _{\theta \theta }= & {} 2\left[ {\displaystyle \frac{l_2 c^2 + l_3s^2 -2l_4cs}{\lambda _z}} + 2\sqrt{\lambda _z} \gamma \left( \left( l_2-l_3\right) cs + l_4\left( c^2-s^2\right) \right) +\lambda _z^2\gamma ^2\left( l_2s^2+l_3c^2+2l_4cs\right) \right] -p \,,\nonumber \\ \sigma _{z\theta }= & {} 2\left[ \sqrt{\lambda _z}\left( \left( l_2-l_3\right) cs +l_4\left( c^2-s^2\right) \right) + \lambda _z^2\gamma \left( l_2s^2+l_3c^2 + 2l_4cs\right) \right] \,, \nonumber \\ \sigma _{zz}= & {} 2 \lambda _z^2\left( l_2s^2 + l_3c^2 + 2l_4cs \right) -p \,, \,\sigma _{rr}= {\displaystyle \frac{2l_1}{\lambda _z}} -p \,, \end{aligned}$$

53 $$\begin{aligned} l_i= & {} \left( {\displaystyle \frac{\partial W}{\partial {\varvec{C}}}}\right) _{ii} \,, \,i=1,2,3 \,, \,l_4 = \left( {\displaystyle \frac{\partial W}{\partial {\varvec{C}}}}\right) _{23} \,. \end{aligned}$$

The normal force ${\mathcal {N}}$ and the torque per unit deformed area ${\mathcal {M}}$ applied at the ends of the cylinder are as follows:

54 $$\begin{aligned} {\mathcal {N}} = 2\pi \int _0^a \sigma _{zz}r \, \textrm{d}r, \quad {\mathcal {M}} = {\displaystyle \frac{2}{a^2}} \int _0^a \sigma _{z\theta }r^2 \, \textrm{d}r \,, \,a={\displaystyle \frac{A}{\sqrt{\lambda _z}}} \,. \end{aligned}$$

To remove the hydrostatic pressure term in (54)$_1$, we use the equilibrium relation

55 $$\begin{aligned} \sigma _{rr}+\sigma _{\theta \theta } = \frac{1}{r}\frac{\textrm{d}\left( r^2\sigma _{rr}\right) }{\textrm{d}r} \,. \end{aligned}$$

and reformulate (54)$_1$ in the form

56 $$\begin{aligned} {\mathcal {N}} = \pi \int _0^a (2\sigma _{zz}-\sigma _{rr}-\sigma _{\theta \theta }) r \, \textrm{d}r \,. \end{aligned}$$

**Figure 7:**
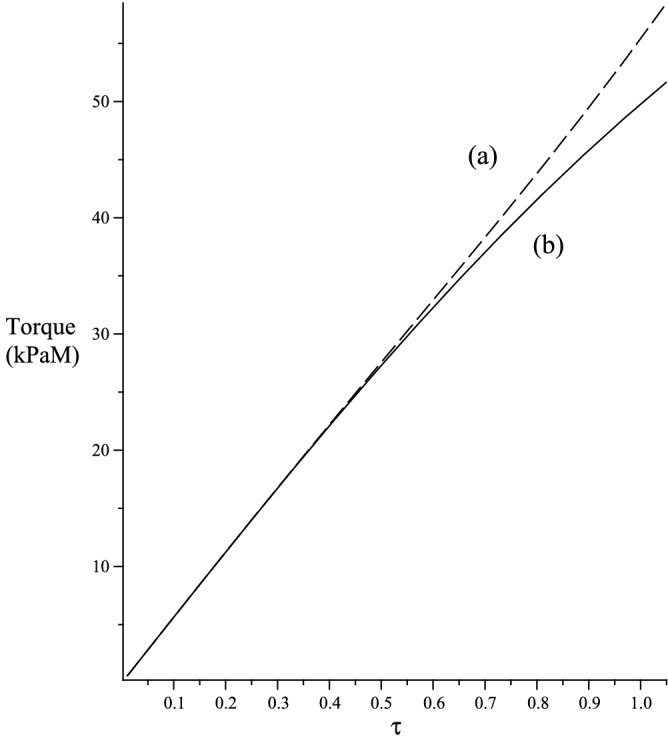
Torque, ${\mathcal {M}}$ versus $\tau$. (**a**) Elastic solid with fibre bending stiffness. (**b**) Elastic solid with no fibre bending stiffness. $\lambda _z=1.5$.

It is clear from Fig. 7 that, for an axial stretch $\lambda _z=1.5$, we require more torque to twist an elastic solid cylinder with fibre bending stiffness.

## Conclusion

We have modelled elastic resistance due to changes in the curvature of the fibres without using the second gradient theory. In view of this, the proposed hyperelastic model is simple and does not contain couple stresses (which is required in a second gradient model). Hence, the proposed model is more realistic in the sense that a carbon fiber reinforced polymer is a non-polar material, where couple stresses do not exist. In the near future, FEM solutions of the proposed model will be obtained and we will extend this model to polymers that are reinforced with a family of two fibres.

## Supplementary Information

Supplementary Information.

## Data Availability

All data generated or analysed during this study are included in this published article [and its supplementary information files].
