# Supplementary material for: A non-second-gradient model for nonlinear elastic bodies with fibre stiffness
Source: Sci Rep. 2023 Apr 21;13:6562. doi: 10.1038/s41598-023-33670-6 (PMC10121710; doi:10.1038/s41598-023-33670-6)
Supplement: Supplementary file 1 — Supplementary Information. [file 41598_2023_33670_MOESM1_ESM.pdf]

Appendix A

To ensure physically reasonable responses, restrictions are imposed on the proposed strain energy function which in turn restrict the values of the material constants. To obtain these restrictions, without loss of generality, we let $\boldsymbol{a}$ and $\boldsymbol{k}$, at any point $\boldsymbol{x}$, to have the Cartesian components $[1,0,0]^T$ and $[0,1,0]^T$, respectively. Let e_{ij} be the Cartesian components of the infinitesimal strain tensor $\boldsymbol{E}$ and in view of the incompressibility constraint $e_{11}+e_{22}+e_{33}$, the strain energy function takes the form

$$W=\frac{1}{2}[(c_1+2\mu)e_{11}^2+2(\rho^2\kappa_3+2\mu)e_{11}e_{22}+(c_2+2\mu)e_{22}^2+4c_3e_{32}^2+4c_4e_{31}^2+4c_5e_{12}^2]\,, \tag{A1}$$

where $c_1=\kappa_1+2\mu+4\mu_1$, $c_2=\rho^4\kappa_2+2\mu+4\rho^2\mu_2$, $c_3=\mu+\rho^2\mu_2$, $c_4=\mu+\mu_1$, $c_5=\mu+\mu_1+\rho^2\mu_2$. The necessary and sufficient conditions for (A6) to be positive definite are:

$$c_3>0\ \ ,c_4>0\ \ ,c_5>0\ \ ,c_1+2\mu>0$$

$$(c_1+2\mu)(c_2+2\mu)>(\rho^2\kappa_3+2\mu)^2\,. \tag{A2}$$

Below we derive, for finite strain deformations, sufficient conditions for the strain energy W to be positive definite. Consider the tensor $\boldsymbol{S}$ (coaxial with the right stretch tensor $\boldsymbol{U}$) with the eigenvalues $s(\lambda_i)$, i.e.,

$$\boldsymbol{S}=\sum_{i=1}^{3}s(\lambda_i)\boldsymbol{u}_i\otimes\boldsymbol{u}_i\,. \tag{A3}$$

Hence, we have

$$\boldsymbol{S}^2=\sum_{i=1}^{3}s^2(\lambda_i)\boldsymbol{u}_i\otimes\boldsymbol{u}_i\,.$$

For simplicity, we only derive sufficient conditions for $r_1=r_2=\ldots=r_7=s$ The strain energy then takes the form

$$\begin{gathered}W=\mu tr(\boldsymbol{S}^2)+2\mu_1\boldsymbol{a}\cdot\boldsymbol{S}^2\boldsymbol{a}+2\rho^2\mu_2\boldsymbol{k}\cdot\boldsymbol{S}^2\boldsymbol{k}\\+\frac{\kappa_1}{2}(\boldsymbol{a}\bullet\boldsymbol{S}\boldsymbol{a})^2+\rho^4\frac{\kappa_2}{2}(\boldsymbol{k}\cdot\boldsymbol{S}\boldsymbol{k})^2+\rho^2\kappa_3(\boldsymbol{a}\cdot\boldsymbol{S}\boldsymbol{a})(\boldsymbol{k}\cdot\boldsymbol{S}\boldsymbol{k})\,.\end{gathered} \tag{A4}$$

if we let $\boldsymbol{a}$ and $\boldsymbol{k}$ have the Cartesian components $[1,0,0]^T$ and $[0,1,0]^T$, respectively, we then have

$$W=\frac{1}{2}\boldsymbol{s}_m^T\boldsymbol{A}_m\boldsymbol{s}_m\,, \tag{A5}$$

where $\boldsymbol{s}_m=[S_{11},S_{22},S_{33},2S_{32},2S_{31},2S_{12}]^T$ and S_{ij} are the Cartesian components of $\boldsymbol{S}$,

$$\boldsymbol{A}_M=\begin{bmatrix}c_1 & \rho^2\kappa_3 & 0 & 0 & 0 & 0\\ \rho^2\kappa_3 & c_2 & 0 & 0 & 0 & 0\\ 0 & 0 & 2\mu & 0 & 0 & 0\\ 0 & 0 & 0 & c_3 & 0 & 0\\ 0 & 0 & 0 & 0 & c_4 & 0\\ 0 & 0 & 0 & 0 & 0 & c_5\end{bmatrix}\,, \tag{A6}$$

We note that, due to the incompressibility constraint $\lambda_1\lambda_2\lambda_3 = 1$, some of the S_{ij} are not independent and, in view of this, necessary and sufficient conditions for W to be positive definite are not trivial to obtain. However, if all the eigenvalues of the matrix $\boldsymbol{A}_m$ are positive then W_e in (A5) is positive definite. Hence, sufficient conditions for positive definite W are:

$$c_1 > 0\,, \quad c_1c_2 > \rho^2\kappa_3^2\,, \quad \mu > 0\,, \quad c_3 > 0\,, \quad c_4 > 0\,, \quad c_5 > 0\,. \tag{A7}$$

Appendix B

Let $\boldsymbol{d}_\alpha$, $\alpha = 0, 1, \ldots$ be approximate values of $\boldsymbol{d}$ that are obtained via the description below. If the deformation is not known, as a first iteration, we first solve the boundary value problem (BVP) using the strain energy

$$W = W_{(T)} \tag{B1}$$

and the solution of this boundary value problem is used to evaluate the first approximation $\boldsymbol{d}_0$. We then solve the BVP via the following iteration:
For $i = 0, 1, \ldots$
Solve the BVP using $\boldsymbol{d}_i$ and the strain energy function

$$W = W_{(T)} + W_{(\Lambda)}\,. \tag{B2}$$

Obtain $\boldsymbol{d}_{i+1}$ from the solution of the BVP.
If $\|\boldsymbol{d}_{i+1} - \boldsymbol{d}_i\| <$ tolerance. Stop. We consider this is the final solution,
else
Continue with the iteration
endif
Note that, $\| \bullet \|$ is the Euclidean norm and we assume that the above iteration converges.
